# Do school teachers and primary contacts in residential youth care institutions recognize mental health problems in adolescents?

**DOI:** 10.1186/s13034-016-0109-4

**Published:** 2016-06-29

**Authors:** Anne Mari Undheim, Stian Lydersen, Nanna Sønnichsen Kayed

**Affiliations:** Faculty of Medicine, Regional Centre for Child and Youth Mental Health and Child Welfare—Central Norway, Norwegian University of Science and Technology (NTNU), PB 8905, MTFS, 7491 Trondheim, Norway

**Keywords:** Mental health, Adolescents, Residential youth care, Primary contacts, Teachers

## Abstract

**Background:**

Studies show that adolescents living in residential youth care (RYC) institutions experience more mental health problems than others. This paper studies how well teachers and primary contacts in RYC institutions recognize adolescents’ mental health problems as classified by The Child and Adolescent Psychiatric Assessment diagnostic interviews (CAPA).

**Methods:**

All residents between 12 and 23 years of age living in RYC institutions in Norway and enrolled in school at the time of data collection were invited to participate in the study. Of the 601 available children, 400 participated in the study, namely 230 girls, mean age = 16.9 years, SD = 1.2 and 170 boys, mean age = 16.5 years, SD = 1.5. The Child Behavior Checklist (CBCL) and Teacher’s Report Form (TRF) were used. The sensitivity and specificity of these instruments were studied.

**Results:**

We observed a significant gap between the mental health problems diagnosed by the CAPA interviews and the problems reported by primary contacts on the CBCL and by teachers on the TRF. The CBCL showed a higher sensitivity than the TRF, whereas the TRF showed a higher specificity than the CBCL. Both primary contacts and teachers classified externalizing problems fairly well such as ADHD in both genders and conduct disorder in girls. Both teachers and primary contacts, however, had more problems detecting internalizing problems. Teachers may have a tendency to view most students as healthy and to underestimate the severity of their problems, whereas primary contacts may tend to overestimate the number of problems and view adolescents as sicker than they really are.

**Conclusion:**

The Child Welfare System should revise their intake procedures to detect possible problems early on and to introduce the necessary treatment. It is important to identify factors that increase healthy school adaption in order for these adolescents to accomplish school in a proper way since education is important for a successful adult life.

## Background

Compared with other children, adolescents in contact with the Child Welfare System (CWS) tend to be less successful later in life across a wide range of areas [1, 2]. These adolescents experience problems with mental health, drug addiction, crime, poor education, and unemployment [3, 4]. According to Harpin et al. [5], out-of-home youth in Ireland had greater risks (suicidal risk, mental health distress) and fewer protective factors (feeling that parents and other adults care about them and a sense of school connectedness) than those in the comparison group. Several studies have confirmed that CWS clients have more mental health problems than others [6, 7]. A recent Norwegian study reported that 76.2 % of the youth living in residential youth care (RYC) in Norway fulfilled the symptoms, onset, duration and impairment criteria for at least one DSM-IV diagnosis. That study reported higher prevalence rates for depressive and anxiety psychiatric disorders than for behavioral disorders [8]. The most frequent diagnoses or diagnostic categories observed were depression and dysthymia (37.3 %), followed by any anxiety disorder (34.9 %), Attention Deficit Hyperactivity Disorder (ADHD) (32.3 %) and Asperger’s Syndrome (AS) (23.2 %); however, only 37 % reported receiving help for these diagnoses. According to Levitt [9], there is a significant gap between children in the CWS population who need services and children who receive services, as the majority of child welfare agencies do not systematically screen children in the CWS for mental health problems.

Adolescents who have been removed from home because of a lack of adequate parental care rarely have access to consistent educational support, which is a resource that is taken for granted by most adolescents who live with their parents [10]. There is no reason to think that adolescents in RYC are better off in this area. In general, adolescents in out-of-home care are at a high risk of having poor educational outcomes [11–13], and they have lower rates of school attendance [14], more cases of drop-outs [15] and lower grades compared to children living at home [16].

School failure is one of the more serious negative outcomes for young people in CWS. International studies have consistently shown that they score significantly below their peers on a range of school outcome measures [17]. In a Norwegian study by Clausen and Kristofersen [12], 35 % of former CWS clients had completed high school, compared to 80 % of a non-client sample. Similar results have also been found in Sweden [13]. Jaffee and Gallop [18] found that relatively few CWS adolescents (approximately 40 %) function normally in school and that even fewer are resilient across several domains, i.e., school achievement, mental health, and social competence. In addition, caregivers’ attitudes towards school may influence children’s success in school [19]. Marginalization and social exclusion are considered to be outcomes of a lack of coping in school [20, 21], as education plays a major role in an individual’s ability to successfully settle into adult life.

It has also been reported that adolescents’ secondary school careers are negatively affected by the presence of acute psychosocial health problems [22]. Kessler et al. [23] have reported that in the United States, adolescents with psychiatric disorders account for 14.2 % of high school dropouts. Furthermore, externalizing problems are reported to impair educational attainment [24]. Poor educational attainment has also been found to predict the onset of schizophrenia spectrum disorders [25].

Adolescents in RYC do not have parents to attend to their needs, and they depend more on other people, for example on primary contacts in RYC institutions or teachers, to disclose their problems. Adolescents spend a substantial amount of time in school, and it is therefore important for teachers to help detect serious problems. However, because of residential instability, adolescents in care tend to experience multiple school transfers [26], which makes it difficult for teachers to observe symptoms over time. On the other hand, for some adolescents in RYC, teachers may be among the more stable persons in their life.

Studies have shown that teachers in Scandinavia generally report relatively low levels of emotional/behavioral problems among school-aged children [27]. However, we do not know how well teachers detect mental health problems in children living in RYC, who, according to studies, suffer from far more mental health problems than the general population [6].

Teachers are reported to be more accurate in identifying children who are at risk of externalizing disorders than those at risk of internalizing disorders [28]. Internalizing problems such as feelings of depression or loneliness are presumably less observable and depend more on interpretation by informants than externalizing problems such as fighting or teasing. Recognizing mental health problems, however, is very important among adolescents living in RYC, in which more than 70 % of adolescents have been found to meet the criteria for at least one psychiatric disorder [8, 29].

Previous studies on teacher’s reports of adolescent mental health problems most often included adolescents living with families and often focused on younger children. To our knowledge, no studies have focused on adolescents living in RYC institutions and their situations at school. Earlier studies tended to include children in the CWS system in general.

The aim of the present study which is part of a larger study on adolescents in RYC was to explore whether mental health problems, as assessed by The Child and Adolescent Psychiatric Assessment (CAPA) [30], among adolescents living in RYC institutions were detected by primary contacts at their institutions and their teachers. The research question was whether adolescents’ internalizing (affective and anxiety) disorders, conduct disorder (CD) and ADHD problems as reported by teachers and primary contacts were consistent with the diagnostic categories identified in CAPA [30]. As the symptoms of externalizing (CD) problems and ADHD are more easily identified as disruptive, we hypothesized that teachers and primary contacts would more easily detect these two categories than internalizing problems.

## Method

### Participants

**Fig. 1:**
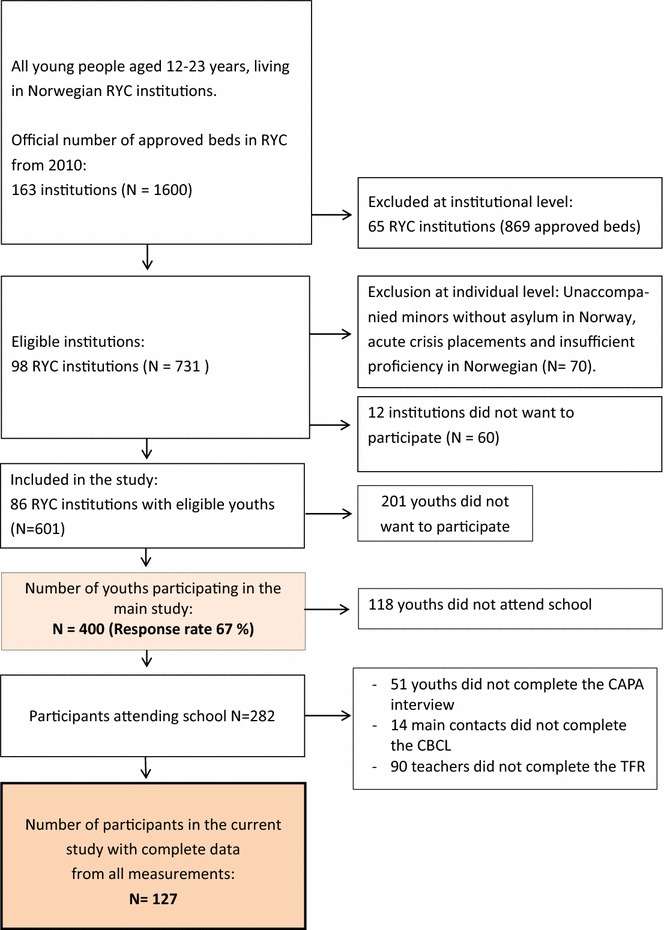
Flowchart of number of participants in the study

All residents between 12 and 23 years of age living in RYC institutions in Norway and enrolled in school at the time of data collection were invited to participate in the study. The age group 12–23 was chosen because that was the age group available in the RYC institutions. Unaccompanied minors without asylum in Norway and youth placed in acute care were considered to be in such a high state of crisis that collecting their data was not prioritized, and they were therefore excluded from the study, see flowchart of the study, Fig. 1. Youth who lacked sufficient proficiency in Norwegian to be interviewed were also excluded. For more details about the sample, see Jozefiak et al. [8].

### Setting

RYC institutions in Norway are organized by The Norwegian Directorate for Children, Youth and Family under the Ministry of Children and Equality. The directorate is responsible for all RYC institutions, but the institutions can be both publicly and privately owned. A Norwegian RYC institution is typically a small unit (3–5 residents) in which youth are encouraged to live as close to a normal life as possible, attending school and participating in leisure activities.

The CWS decide, as part of their intake procedures, what kind of care is best suited for each child, mostly foster care or RYC. For older children it is more difficult to find foster care so adolescents are mostly placed in RYC. It differs how long the children stay in RYC. Intentionally they stay as short as possible, however, for some their home situation is not good enough for moving back. Most of the children have contact with their biological families during the stay.

At the institutions each child is assigned a primary care giver among the available RYC staff during the stay. The RYC staff often holds a bachelor degree in social, health or pedagogical areas, however, about a third of the staff is without higher education. The work of the staff is based on a milieu therapeutic model and shows a generally limited knowledge of psychiatric diagnosis and treatment.

### Procedures

A database of all RYC institutions in Norway was created by the project team based on information from The Norwegian Directorate for Children, Youth and Family Affairs. The RYC institutions were randomly selected and contacted in a random order. Data collection was conducted by four trained research assistants in the RYC institutions between June 2011 and July 2014 and lasted approximately 4 h per youth. Due to the length of CAPA and the adolescents’ challenges related to concentration and stamina, not all residents were able to complete the psychiatric interview. The child’s primary contact reported on each resident’s mental health problems using The Child Behavior Checklist (CBCL) [31]. The adolescents in RYC attended the local schools. All students are assigned to a homeroom teacher who has a special responsibility for the adolescent in school, including filling in forms and offering student-parents meetings minimum twice a year. This teacher collects information from other teachers about subjects other than his own. The person at school working closest with the child (homeroom teacher or teacher assistant) filled out the Teacher’s Report Form (TRF) [32].

The few participant 19 years old (N = 5; 1.8 %) were assessed with the Achenbach System of Empirically Based Assessment (ASEBA) 11–18 year versions [31]. This was assumed to give more similar and comparable information across age-groups than using another instrument for the oldest.

Participants were recruited using procedures approved by the Norwegian Regional Committee for Medical and Health Research Ethics, and written consent was obtained. The parents have the custody of adolescents when the placement is voluntary, and the CWS service has the custody of adolescents placed involuntary. Informed written consent was signed by the adolescents regardless of their age. According to the Norwegian Health Research Legislation at the age of 16, the adolescents are considered old enough to sign their own consent. For adolescents under the age of 16, written consent was also provided by parents or CWS.

### Measures

Achenbach et al. [33] constructed several measures within their package Achenbach System of Empirically Based Assessment (ASEBA). Three of those were used in the present study: CBCL, TRF, and CAPA. We have not found any studies reporting on the associations between The Child and Adolescent Psychiatric Assessment (CAPA) [30] and The Child Behavior Checklist (CBCL) [31] or the Teacher’s Report Form (TRF) [31, 32]. However, there have been some studies on the associations between scores on the CBCL and TRF. A large study in 21 societies by Rescorla et al. [34] found that CBCL scores were relatively higher than the TRF scores on most scales.

*The Child Behavior Checklist (CBCL)* consists of 118 Likert-type and two open-ended items rated on a 0–2 scale (0 = not true, 1 = somewhat or sometimes true, or 2 = very true or often true). For the present study, we used the following eight syndrome scales from the 2001 version [31] of the checklist for children and adolescents aged 6–18 years: Anxious/depressed, Withdrawn/depressed, Somatic complaints, Social problems, Thought problems, Attention problems, Rule-breaking behavior and Aggressive behavior. The Norwegian version of the CBCL has shown satisfactory reliability and validity (alphas of 0.93, 0.84 and 0.89 for the total scale and Internalizing and Externalizing subscales, respectively) [35]. According to the Multicultural Supplement to the ASEBA manual [36], Norway is included in Group 3, and the norms and cut-offs were set according to this group; see Table 1.

**Table 1:** Cut-offs of the different diagnoses according to the cultural norm in the Multicultural Supplement to the manual for the ASEBA school-age forms and profiles

	Boys		Girls	
	Borderline	Clinical range	Borderline	Clinical range
CBCL category (cultural norm 1)				
Affective problems	5	8	5	8
Anxiety problems	3	5	4	5
Conduct problems	5	8	5	8
ADHD problems	7	10	6	8
TRF category (cultural norm 2)				
Affective problems	7	9	5	8
Anxiety problems	3	5	3	5
Conduct problems	9	14	5	10
ADHD problems	17	22	12	17

#### Teacher’s Report Form (TRF) [32]

To date, this is one of the most used measures of emotional/behavioral problems in school. The TRF consists of teacher’s ratings of a child’s academic performance, adaptive characteristics and conduct problems. Teachers were asked to rate the degree of a child’s emotional and behavioral problems during the previous 2 months on a 0–2 scale (0 = not true as far as they know; 1 = somewhat or sometimes true; 2 = very true or often true).

The scale consisted of 118 problem items plus 2 open-ended items (not used here). Total problems scores thus range from 0 to 236. The TRF has been found to have internal consistency in 21 countries with a strong construct validity alpha [34, 36]. According to the Multicultural Supplement to the ASEBA manual [36], Norway is included in Group 3, and the norms and cut-offs were set according to this group; see Table 1.

#### The Child and Adolescent Psychiatric Assessment (CAPA)

The CAPA is an interviewer-based semi-structured psychiatric interview that collects data on the onset dates, duration, frequency, and intensity of symptoms of a wide range of psychiatric diagnoses according to the DSM-IV [30]. The interview serves as a guide to determine whether a symptom is present at pre-specified levels, and the interviewer is expected to probe until she or he can decide whether the symptom is present. Information concerning the frequency, onset, intensity and duration is obtained. Moreover, functional impairment is captured. The test–retest reliability of the assessment has been shown to be adequate [30]. Interviewers (N = 4) had at least a bachelor’s degree in a relevant field and extensive experience working with children and families. The inter-rater reliability of the rater pairs as estimated by Gwet’s AC1 (and agreement rate) ranged between 0.74 and 1.0, except for substance abuse, which had an AC1 of 0.69. Gwet’s AC1 was calculated in AgreeStat (supplied commercially by Gwet at http://www.agreestsat.com/agreestat.html) [8].

### Statistics

Throughout this study, we considered the diagnoses from the CAPA interview as the diagnostic standard. First, we studied the sensitivity and specificity of the CBCL and TRF for each diagnosis of the diagnostic groups. In this context, a CBCL score equal to or above the gender-specific borderline cut-off value in Table 1 was regarded as a positive CBCL, and the same method was applied for the TRF.

Second, we conducted ROC (receiver operating diagnostic curve) analyses. When different cut-off values are used for the CBCL (or TRF) scores, different pairs of sensitivity and specificity values emerge. An ROC curve connects these paired values of specificity and sensitivity. The area under the ROC curve, AUC, is a measure of the ability of the value to discriminate between clinical cases and non-clinical cases. The AUC equals 1 if there is perfect discrimination, and an AUC of 0.5 indicates discrimination that is no better than chance. We regarded an AUC below 0.7 as poor, between 0.7 and 0.8 as acceptable, between 0.8 and 0.9 as excellent, and above 0.9 as outstanding discrimination, as recommended by Hosmer et al. [37]. One interpretation of the AUC is as follows: if one randomly picks a diseased individual and a non-diseased individual, the AUC is the probability that the diseased individual scores higher than the non-diseased individual on the scale. The statistical analyses were conducted using SPSS 22 and Stata 13. A two-sided p value <0.05 indicated statistical significance.

## Results

### Attrition

Of the 601 available children, 201 refused participation, representing an attrition rate of 33 %. Thus 400 children participated in the study, consisting of 230 girls with a mean age of 16.9 years, SD = 1.2, and 170 boys with a mean age of 16.5 years, SD = 1.5. In total, 86 (of 98 eligible) institutions participated, resulting in a response rate of 88 % [8]. The demographic infomation about the sample attending school is shown in Table 2.

**Table 2:** Demographic information of the sub-sample attending school (n = 282)

Demographics	n	%
Boys	132	46.8
Girls	150	53.2
Age in years		
12–15	99	35.1
16–18	178	63.1
19	5	1.8
Prevalence of diagnostic categories from Child and Adolescent Psychiatric Assessment (CAPA)		
Affective disorders	78	27.7
Anxiety disorders	77	27.3
ADHD	90	31.9
Conduct disorder	37	13.1

### Sensitivity and specificity

The comparisons between the CAPA diagnosis and the CBCL scores in the four diagnostic categories showed a sensitivity ranging from 0.60 to 0.82, whereas the TRF showed a sensitivity ranging from 0.39 to 0.54; see Table 3. The primary contacts’ reports consistently showed a higher sensitivity across all diagnostic categories compared with those of the teachers. The opposite pattern was found when comparing specificity. The agreement between the CAPA diagnosis in the four diagnostic categories and the CBCL showed a specificity ranging from 0.30 to 0.74, whereas the TRF showed a specificity ranging from 0.70 to 0.88. The primary contacts’ reports had a consistently lower specificity across all diagnostic categories than those of the teachers, see Table 3.

**Table 3:** Cross-tables of results from the CAPA interview, set as the “gold standard,” indicating psychiatric diagnosis in the four categories, affective, anxiety, and conduct disorder, and ADHD, compared to reports on the CBCL and TRF corresponding to DSM-oriented scales

CAPA—diagnostic categories		CBCL—corresponding DSM-oriented scales					CAPA—diagnostic categories		TRF—corresponding DSM-oriented scales				
		Yes	No	Total	Estimate				Yes	No	Total	Estimate	
Total sample													
Affective disorder	Yes	61	13	74	Sensitivity	0.82	Affective disorder	Yes	22	27	49	Sensitivity	0.45
	No	69	70	139	Specificity	0.50		No	26	66	92	Specificity	0.72
	Total	130	83	213				Total	48	93	141		
Anxiety disorder	Yes	45	29	74	Sensitivity	0.61	Anxiety disorder	Yes	22	28	50	Sensitivity	0.44
	No	55	84	139	Specificity	0.60		No	27	64	91	Specificity	0.70
	Total	100	113	213				Total	49	92	141		
Conduct disorder	Yes	25	7	32	Sensitivity	0.78	Conduct disorder	Yes	15	13	28	Sensitivity	0.54
	No	127	54	181	Specificity	0.30		No	29	85	114	Specificity	0.75
	Total	152	61	213				Total	44	98	142		
ADHD	Yes	40	27	67	Sensitivity	0.60	ADHD	Yes	23	36	59	Sensitivity	0.39
	No	38	108	146	Specificity	0.74		No	10	73	83	Specificity	0.88
	Total	78	135	213				Total	33	109	142		
BOYS													
Affective disorder	Yes	14	6	20	Sensitivity	0.70	Affective disorder	Yes	5	9	14	Sensitivity	0.36
	No	34	40	74	Specificity	0.54		No	9	44	53	Specificity	0.83
	Total	48	46	94				Total	14	53	67		
Anxiety disorder	Yes	17	9	26	Sensitivity	0.65	Anxiety disorder	Yes	10	8	18	Sensitivity	0.56
	No	29	39	68	Specificity	0.57		No	9	40	49	Specificity	0.82
	Total	46	48	94				Total	19	48	67		
Conduct disorder	Yes	15	6	21	Sensitivity	0.71	Conduct disorder	Yes	7	12	19	Sensitivity	0.37
	No	51	22	73	Specificity	0.30		No	6	43	49	Specificity	0.88
	Total	37	57	94				Total	13	55	68		
ADHD	Yes	19	12	31	Sensitivity	0.61	ADHD	Yes	9	20	29	Sensitivity	0.31
	No	12	51	63	Specificity	0.81		No	2	37	39	Specificity	0.95
	Total	31	63	94				Total	11	57	68		
GIRLS													
Affective disorder	Yes	47	7	54	Sensitivity	0.87	Affective disorder	Yes	17	18	35	Sensitivity	0.49
	No	35	30	65	Specificity	0.46		No	17	22	39	Specificity	0.56
	Total	82	37	119				Total	34	40	74		
Anxiety disorder	Yes	28	20	48	Sensitivity	0.58	Anxiety disorder	Yes	12	20	32	Sensitivity	0.38
	No	26	45	71	Specificity	0.63		No	18	24	42	Specificity	0.57
	Total	54	65	119				Total	30	44	74		
Conduct disorder	Yes	10	1	11	Sensitivity	0.91	Conduct disorder	Yes	8	1	9	Sensitivity	0.89
	No	76	32	108	Specificity	0.30		No	23	42	65	Specificity	0.65
	Total	86	33	119				Total	31	43	74		
ADHD	Yes	21	15	36	Sensitivity	0.58	ADHD	Yes	14	16	30	Sensitivity	0.47
	No	26	57	83	Specificity	0.69		No	8	36	44	Specificity	0.82
	Total	47	72	119				Total	22	52	74		

Sensitivity and specificity are reported for the four diagnostic groups for both CBCL and TRF. The table presents results for the total sample and for boys and girls separately

For boys, as shown in the middle panel of Table 3, the agreement between a CAPA diagnosis in the four diagnostic categories and the primary contacts’ report on the CBCL showed a sensitivity ranging from 0.61 to 0.81. The agreement between a CAPA diagnosis in the four diagnostic categories and the teachers’ report on the TRF showed a sensitivity ranging from 0.31 to 0.56. The CBCL consistently showed a higher sensitivity across all diagnostic categories than the TRF. The opposite pattern was found when comparing specificity. Primary contacts’ reports on the CBCL showed a specificity ranging from 0.54 to 0.81. Teachers’ reports on the TRF showed a specificity ranging from 0.83 to 0.95. The CBCL completed by the primary contacts consistently showed a lower specificity across all diagnostic categories compared with the TRF completed by the teachers.

For girls, as observed in the bottom panel of Table 3, the agreement between a CAPA diagnosis in the four diagnostic categories and the primary contacts’ report on the CBCL showed a sensitivity ranging from 0.59 to 0.96. The agreement between a CAPA diagnosis in the four diagnostic categories and the teachers’ report on the TRF showed a sensitivity ranging from 0.37 to 0.89. The primary contacts’ reports consistently showed a higher sensitivity across all diagnostic categories compared with those of the teachers. The opposite pattern was found when comparing specificity. Primary contacts’ reports on the CBCL showed a specificity ranging from 0.31 to 0.69. Teachers’ reports on the TRF showed a specificity ranging from 0.56 to 0.79. For all diagnostic categories, except for the CAPA Anxiety subscale, the CBCL completed by the primary contacts showed a consistently lower specificity across all diagnostic categories than the TRF completed by the teachers.

### ROC

**Fig. 2:**
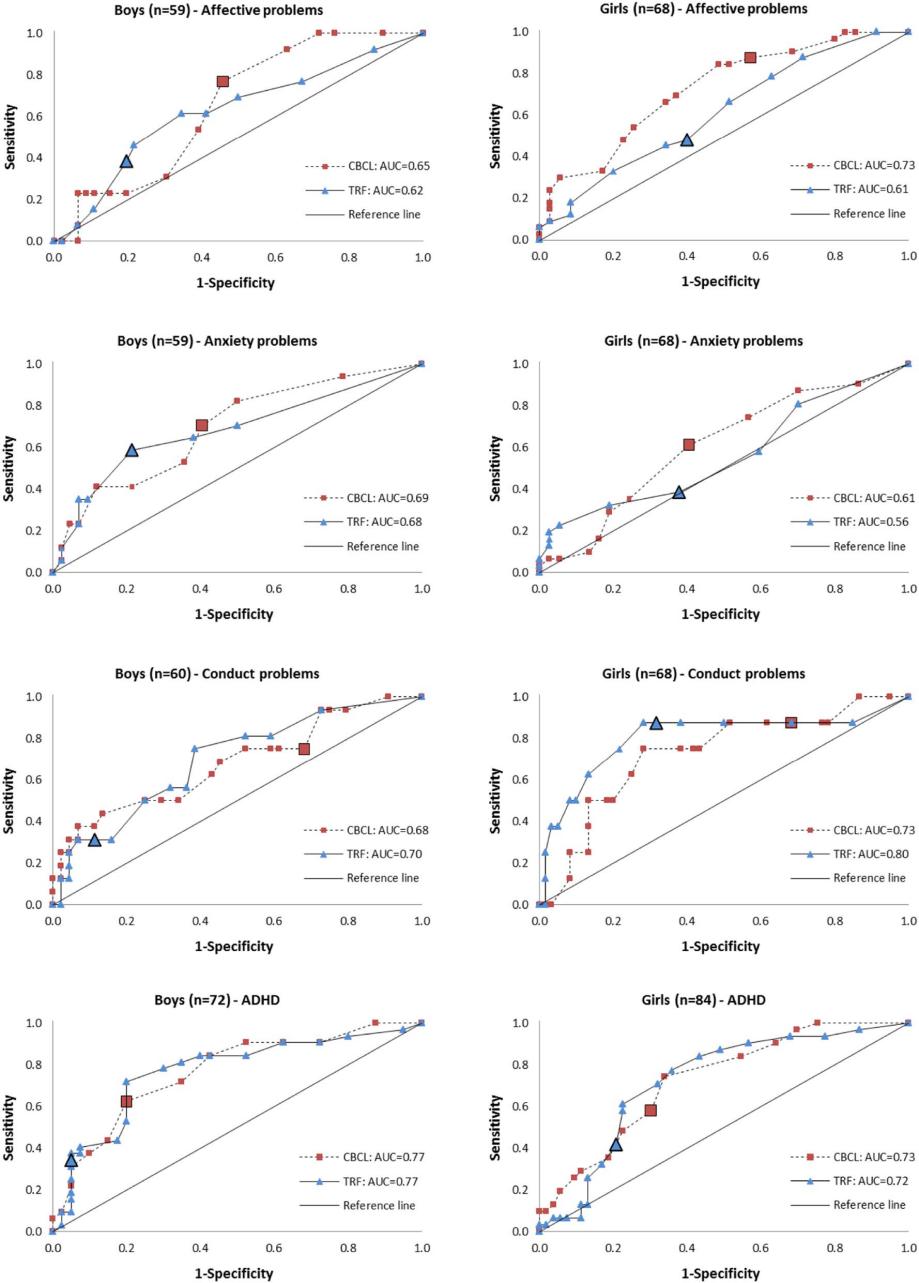
ROC curves (receiver operating diagnostic curve) connecting corresponding values of sensitivity and specificity for the CBCL and TRF with the CAPA diagnostic standard. The enlarged symbols (*square* and *triangle*) represent the sensitivity and specificity obtained using the defined cut-off values in Table 1

Figure 2 shows the ROC with the corresponding sensitivity and specificity for all possible cut-off values, for each of the diagnostic categories, for the CBCL and the TRF, and separately for boys and girls. In all four diagnostic categories and in both genders, the ROC curves for the CBCL and TRF were quite similar, indicating that the CBCL and TRF scales discriminated between adolescents equally as well. In fact, there were no significant differences between any of the eight pairs of CBCL and TRF comparisons (p values from 0.16 to 0.98). Both primary contacts’ reports on the CBCL and teachers’ reports on the TRF identified ADHD acceptably well in both genders and CD in girls, with AUCs between 0.7 and 0.8. Both primary contacts’ and teachers’ reports poorly detected affective disorders and anxiety in both genders and CD in boys, with AUCs below 0.7. One exception was the CBCL for affective disorders in girls, with an AUC = 0.73.

## Discussion

This paper focused on how well teachers and primary contacts in RYC institutions identified adolescents’ mental health problems as determined by the CAPA diagnostic interview. We observed that there was a significant gap between the mental health problems diagnosed by the CAPA interviews and the problems reported by the primary contact on the CBCL and by the teachers on the TRF. In general, we observed that the reports from the primary contacts showed a higher sensitivity across all diagnostic categories than those of the teachers. This indicates that when adolescents do have a psychiatric disorder, primary contacts are better at identifying this than teachers. Teachers’ ratings showed a higher specificity across all diagnostic categories than those of primary contacts. This indicates that when adolescents do not have a psychiatric disorder, teachers are better at recognizing this than the primary contacts. Ideally, when using any screening instrument, both sensitivity and specificity should be high, thus ensuring that the right individuals are identified with a diagnosis and avoiding overdiagnosis.

As adolescents living in RYC institutions do not have parental educational support, it is up to the institutional staff to meet this need. It is important to identify factors that facilitate healthy adaptation to school for these adolescents to succeed in school. Improving mental health problems is one of these factors, as mental health issues are an important barrier to learning [24]. Teachers were quite good at identifying adolescents who did not meet the criteria for a diagnosis (specificity). This suggests that teachers have a tendency to view these students as healthy and may underestimate the severity of their problems.

On the other hand, adolescents’ primary contacts may overestimate the prevalence of mental health problems and view individuals as more sick than they are. A disadvantage of this perspective is that adolescents may be treated as sick when they are not; they may thus be given fewer responsibilities and fewer demands instead of being encouraged to push themselves and do their best. Underestimating these adolescents and feeling sorry for them because of their previous challenges and experiences is understandable. However, they, more than others, need people to believe in them, encourage them to work, and believe in their ability to succeed. In addition, caretakers’ attitudes towards school have been reported as being important for children to succeed in school [19]. In general, studies have reported that adolescents in out-of-home care are at a high risk for poor educational outcomes [11, 13]. This is a serious problem, as we also know that education plays a major role in successful adult life [38]. Enhancing the educational performance of young people in RYC requires daily and pervasive educational support and encouragement [39]. Both teachers and care persons should work together towards these goals.

Primary contacts were quite good at identifying adolescents who had a diagnosis (sensitivity), whereas teachers had a tendency to underestimate these problems, which is consistent with results from Larsson and Drugli [27] on school-aged children. Adolescents in RYC are dependent on caretakers and teachers to identify their problems. The present study shows that primary contacts detect adolescents’ mental health problems to a certain degree. However, as very few of the adolescents from our study report receiving help for their problems from mental health clinics (37 %, [8]), the institutions do not seem to refer these adolescents to treatment. If RYC personnel or teachers do not act, these adolescents will not receive the help that they need as few adolescents call on medical or other services themselves if they suffer anxiety or feel depressed. This situation is noteworthy. The availability of psychiatric services for this group is further discussed in another paper from the same study [8].

With CBCL sensitivities ranging from 0.60 to 0.82 across the four diagnostic categories based on primary contacts’ reports and sensitivities of the TRF ranging from 0.39 to 0.54 according to teachers, many mental health problems may go undetected. The fact that approximately 76 % of the adolescents in the present study qualified for at least one psychiatric DSM-IV diagnosis [8] shows that there is a gap between the true prevalence of mental health problems (CAPA diagnosis) and the problems reported on the CBCL and TRF.

Suffering from mental health problems prevents individuals from fully focusing on schoolwork. It is therefore very important for caretakers and teachers who see these adolescents every day to recognize their mental health problems and refer them to treatment. These adolescents may also need adjustments in school to promote optimal learning. This is important because education is reported to provide better prospects for an individual’s future [40].

Sensitivity and specificity measure the performance of the cut-off values used to identify adolescents within a borderline range. The fact that the CBCL had a higher sensitivity than the TRF, whereas the TRF had a higher specificity than the CBCL, could be explained by the higher cut-off points for the TRF compared to the CBCL as illustrated in the ROC curves. The area under the ROC curve is a measure of the ability of the value to discriminate between clinical cases and non-clinical cases. This area was similar for the CBCL and TRF for all diagnostic groups.

The hypothesis that externalizing problems would be easier to detect was confirmed, consistent with previous literature [28]. Both primary contacts and teachers classified externalizing problems fairly well such as ADHD in both genders and CD in girls, with AUCs between 0.7 and 0.8, which is considered acceptable [37]. It is understandable that externalizing problems are easier to detect, as some symptoms manifest themselves as disturbing elements that can easily be observed because they may interrupt regular activities. Externalizing problems are also reported to impair educational attainment [24]. Furthermore, learning and behavior problems are often related [41], and these adolescents may be at risk for developing behavioral issues if help is not introduced early enough. It is worth noting that several adolescents had externalizing problems that were undetected by caretakers and teachers, and these adolescents also need help.

Both teachers and primary contacts struggled more in recognizing internalizing problems. They poorly identified affective disorders and anxiety in both genders and CD in boys, with AUCs of their corresponding scales below 0.7, which is considered poor [37]. One exception was the use of the CBCL to detect affective disorders in girls, with an AUC = 0.73, indicating that primary contacts detected these problems at an acceptable level. Internalizing problems, such as depression and anxiety, are more hidden and could be concealed in subtle behavior. These problems are often disguised as withdrawal and passive behavior, and staff and teachers could easily think that adolescents with these problems are shy or want to be left alone. However, it is an important problem that serious diagnoses such as depression and anxiety are so modestly detected by caretakers and teachers. Who else is in a position to identify these problems among these adolescents?

### Strengths and limitations

This study is part of a larger study on adolescents in RYC, was based on three different sources of information, and used standardized international measurements. The study is the first one to look at teachers’ and primary care providers’ ability to recognize mental health struggles in adolescents in RYC. We did not have the permission to include the adolescents’ parents as informants, thereby limiting the knowledge about early development and family functioning before placement in RYC. The study has a fairly high participation rate of 67 %. However, limitations could be sample bias as many institutions refused to participate, also many adolescents refused participation or were unable to complete the measures.

## Conclusion

In the present study, there was a mismatch between the DSM-IV diagnoses among adolescents in RYC and the problems reported by their primary contacts and teachers. Primary contacts’ reports showed a higher sensitivity than those of teachers, whereas the teachers’ TRF scores showed a higher specificity in detecting mental health problems in adolescents in RYC than the primary contacts’ CBCL reports. Both primary contacts and teachers recognized externalizing problems such as ADHD fairly well in both genders as well as CD in girls. Both teachers and primary contacts, however, had more problems detecting internalizing problems. It would be important to create interventions with primary contacts, teachers, and youth to educate and raise awareness about emotional problems. Peer identification is another strategy which also has shown effectiveness amongst youth and might be a resource worth considering in the future. Further studies should investigate this topic more carefully to ensure that these vulnerable adolescents receive sufficient help for their problems. CWS should revise their intake procedures so that possible problems are detected early and that the necessary treatment is introduced.

## Abbreviations

CWS: Child Welfare System
RYC: residential youth care
CAPA: The Child and Adolescent Psychiatric Assessment diagnostic interviews
CBCL: The Child Behavior Checklist
TRF: Teacher’s Report Form
CD: conduct disorder
ADHD: Attention Deficit Hyperactivity Disorder
AS: Asperger’s Syndrome
ROC: receiver operating diagnostic curve
AUC: the area under the ROC curve
